# Achieving universal electrification of rural healthcare facilities in sub-Saharan Africa with decentralized renewable energy technologies

**DOI:** 10.1016/j.joule.2021.09.010

**Published:** 2021-10-20

**Authors:** Magda Moner-Girona, Georgia Kakoulaki, Giacomo Falchetta, Daniel J. Weiss, Nigel Taylor

**Affiliations:** 1 European Commission, Joint Research Centre (JRC), Ispra, Italy; 2 Fondazione Eni Enrico Mattei (FEEM), Milan, Italy; 3 Faculty of Economics and Management, Free University of Bozen-Bolzano, Bolzano, Italy; 4 Curtin University, Bentley, WA, Australia; 5 Telethon Kids Institute, Nedlands, WA, Australia

**Keywords:** healthcare, electricity access, accessibility, solar PV, sustainable development, sub-Saharan Africa, UN Sustainable Development Goals, health facilities, travel time, renewable energy

## Abstract

A potential response to the COVID-19 pandemic in sub-Saharan Africa (SSA) with long-term benefits is to provide electricity for medical equipment in rural health centers and communities. This study identifies a large gap in the electrification of healthcare facilities in SSA, and it shows that decentralized photovoltaic systems can offer a clean, reliable, quick, and cost-effective solution. The cost of providing renewable electricity to each health facility by a stand-alone PV system is analyzed for a given location (incorporating operational costs). The upfront investment cost for providing electricity with PV to >50,000 facilities (mostly primary health posts) currently without electricity is estimated at EUR 484 million. Analysis of the accessibility and population distribution shows that 281 million people could reduce their travel time to healthcare facilities (by an average of 50 min) if all facilities were electrified.

## Highlights

• In rural sub-Saharan Africa, over 50,000 healthcare facilities lack electricity supply
• PV and battery systems offer a clean, reliable, and cost-effective solution
• The estimated investment needed would be just under EUR 500 million
• 281 million people could reduce journey time to electrified facilities by 50 min average

## Context & scale

Our results shed new light on the potential of decentralized energy systems to offer a reliable, quick, and cost-effective way to increase access to electricity for rural healthcare facilities in sub-Saharan Africa. This study identified more than 55,000 such facilities without electricity access and analyzed the costs and benefits of powering each of these with solar photovoltaic and battery storage systems.

Our results can provide a basis for planning electrification programs for health facilities in rural sub-Saharan Africa and can be useful to policy makers, researchers, consultants, and other stakeholders involved in electrification planning and healthcare improvement. The level of granularity, covering community, national, and regional levels, is particularly relevant to prioritizing the allocation of limited governmental funding, highlighting where electrification is most needed and likely to have the greatest impact on health services.

## Graphical abstract

**Figure:**
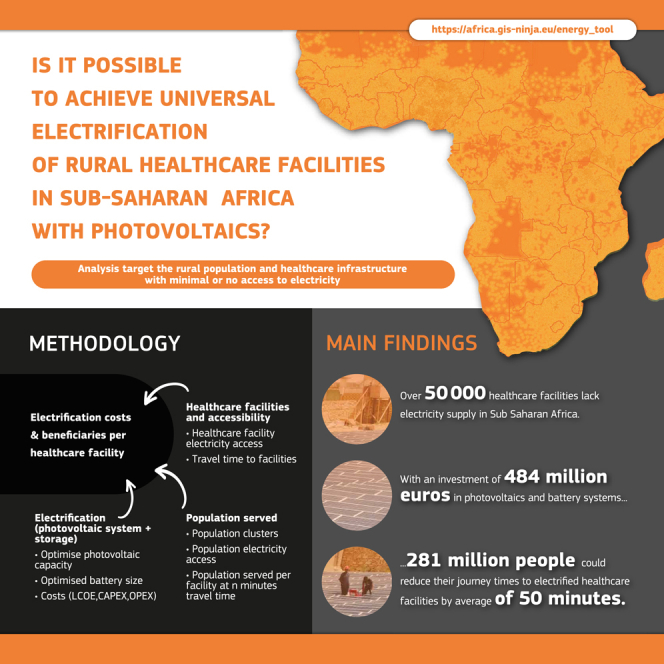

## Teaser

Effective strategies for financing the electrification of healthcare remain a challenge in sub-Saharan Africa. In this study, Moner-Girona et al. identify a large gap in the electrification of healthcare facilities, and they show that decentralized photovoltaic systems can offer a clean, reliable, quick, and cost-effective solution. These findings provide a bottom-up geographic information system (GIS) framework for policy makers, researchers, consultants, and other stakeholders bridging two elements of the sustainable development goals: “energy for all” (SDG7) and “healthcare for all” (SDG3).

## Introduction

**Figure 1:**
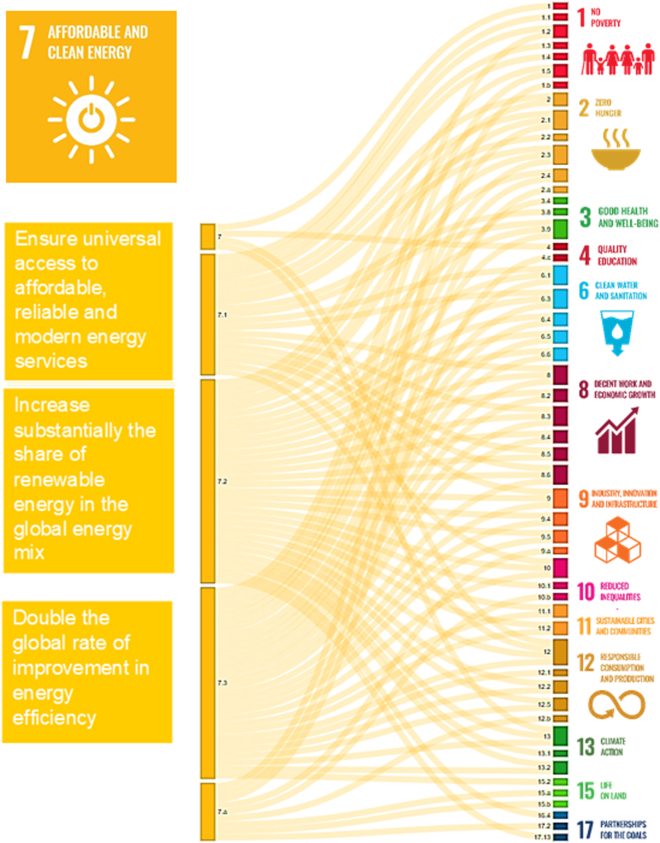
Interlinkages of the sustainable development goal 7 target (SDG.7) “ensure access to affordable, reliable, sustainable, and modern energy for all” with the other sustainable development goal targets Source: adapted from https://knowsdgs.jrc.ec.europa.eu/interlinkages/tools?visualization=chord&edges=0

The United Nations’ (UN) sustainable development goals (SDGs) were adopted by all its member states in 2015 as a universal action call to end poverty, protect the planet, and ensure that all people enjoy peace and prosperity by 2030. For sub-Saharan Africa (SSA), there is a direct link between SDG 7 “Ensure access to affordable, reliable, sustainable, and modern energy” and SDG3 “Good health and wellbeing for all^1^” (Figure 1). Consequently, international organizations such as the World Health Organization (WHO), Global Fund, the United Nations Development Programme (UNDP), the World Bank, USAID, and the EU have prioritized specific programs to support and ensure reliable electricity access to underserved communities in rural areas.^2^^,^^3^

**Figure 2:**
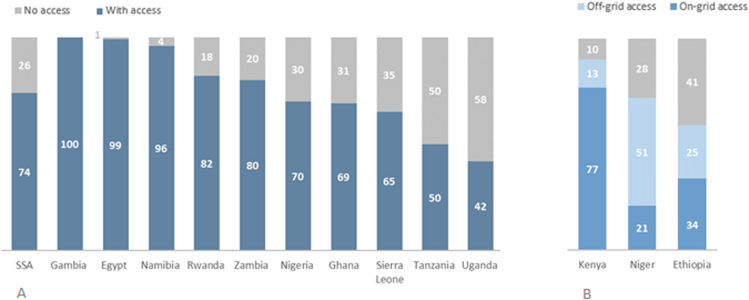
Percentage of healthcare facilities with electricity access in SSA countries (A) Percentage of healthcare facilities with electricity access in selected countries (% of total facilities). (B) Percentage of healthcare facilities (% of total facilities) with on-grid and off-grid electricity access from available country-specific studies (partial field data, 2007–2012). Source: data compilation from IEA et al.,^6^ Adair-Rohani et al.,^7^ Pittalis et al.,^11^ World Bank Group,^12^ UNESCO^13^ and World Bank.^14^

Electricity is a crucial enabler for the provision of healthcare, education, and other services, which in turn can aid communities in achieving socio-economic growth. According to the most recent statistics (2019), there remain 570 million people without access to the most basic electricity services in SSA.^4^ Renewable energy has become the least-cost-effective option for generating electricity in most regions due to institutional, logistical, transport, and last-mile costs in SSA that run well above the global averages.^5^ As a result of these limitations, SSA is not on target to meet SDG7 by 2030.^3^^,^^6^ The lack of energy access extends to healthcare facilities, as one in four facilities lacks a source of electricity, and three out of four facilities lack reliable power.^7^, ^8^, ^9^, ^10^ This situation is most pronounced in rural areas, and it varies considerably between countries (Figure 2). The lack of electrification in large parts of SSA leaves many healthcare facilities with inadequate power for both basic and emergency services.^15^^,^^16^ Electricity is essential for the majority of emergency care activities, including lighting, laboratory tests, and X-rays, as well as the ventilators that are often critically important for respiratory support for COVID-19 patients. Moreover, an estimated 70% of medical devices in the least-developed countries regularly fail or are unavailable, with poor power quality being a major contributing factor.^15^

The COVID-19 pandemic further increased the pressure on the healthcare system in SSA, and it highlighted the importance of energy access for delivering reliable healthcare services. The situation is most challenging for rural communities, which may also have few primary hospitals, shortages of medical staff, poor health literacy, no access to clean water, and poor transportation infrastructure. For such communities, a fast and modular energy solution is urgently needed now, more than ever.^17^ One medium-term approach for boosting resilience in the face of the COVID-19 pandemic in SSA has been to strengthen the critical services by providing power to medical equipment in rural health centers and communities with the help of decentralized renewable energy systems.^18^ Decentralized energy resources (DER) systems typically use renewable energy sources, including small hydro, biomass, biogas, solar, wind, and geothermal power.

The cost of solar photovoltaic (PV) systems has decreased rapidly over the last decade, and due to the expansion of global solar panel manufacturing capacity, solar power costs are now expected to decrease at a faster rate than anticipated before the COVID-19 outbreak.^19^^,^^20^ The costs of lithium-ion battery systems are also falling rapidly.^21^ Installed solar PV power capacity in Africa increased from 273.5 MW_p_ in 2010 to 11 GW_p_ in 2020,^22^ with a corresponding growth in technical capacity and confidence in the technology. Such factors make solar power an attractive option in resource-constrained settings and can make it an affordable option in many communities.^23^^,^^24^ This study focuses on modular solar PV systems (including battery energy storage) as a fast, cost-effective, clean, and reliable solution to supply the power needed in health centers serving Africa’s rural communities.

As shown in Figure 2A, different countries have adapted different strategies for supporting the electrification of healthcare; this heterogeneity is reflected in the percentage of healthcare facilities with electricity access at the country level, which shows large differences across Africa, ranging from almost 100% of healthcare facilities with electricity access in Gambia to 42% in Uganda. Since most sub-Saharan African governments give priority to electrification of social infrastructure (schools and health centers), the majority of countries have higher rates of electrification for health centers than in residential buildings. However, these numbers do not capture other important factors, such as the quality and reliability of the electricity supply. For instance, an average Nigerian household experiences daily power outages for around 19 h.^25^ Electricity access in healthcare facilities in rural SSA has not been thoroughly explored beyond a few country-specific studies with partial field data. Figure 2B distinguishes on-grid and off-grid electricity access for three countries where sufficiently granular data are available, showing the large discrepancies in access possibilities.^6^ In Kenya, 77% of health centers rely on the public national grid for their primary electricity needs. Conversely, in Niger, 51% of health centers use off-grid solutions to cover their primary electricity demand.^6^ Adair-Rohani et al.^7^^,^^26^ found that the proportion of facilities relying only on diesel generators ranged from an average of 33% in Gambia to only 1% in both Uganda and Zambia. Excluding Gambia, 4% of all facilities in SSA, on an average, relied only on diesel generators for electricity.

Irrespective of this evidence, there is a lack of systematic, recently updated data on which facilities are with or without access to electricity and hence a limited awareness of the electrification status. Consequently, it can be difficult to prioritize the provision of reliable electricity when planning and implementing energy investments in the health sector in rural areas. This study aims to tackle this information gap by combining satellite data, national statistical data, and several open-source datasets, as further described in experimental procedures.

Several studies have examined the potential of decentralized renewable systems to power healthcare in developing countries. For instance, Dholakia^27^ discusses the potential and critical barriers to the wider uptake of solar power for electrifying healthcare in developing countries. His review of literature studies shows how power provision enhances healthcare services. The review argues that it is crucial that health policies recognize energy as a critical component of the overall infrastructure. Olatomiwa et al.^28^ illustrated the potential of standalone hybrid renewable energy systems for basic healthcare services in rural areas through an optimization analysis in Nigeria, highlighting the untapped potential and the noteworthy reliability gains, even compared with an unreliable national grid. Franco et al.^29^ carried out a review of sustainable energy access and technologies for healthcare facilities in the Global South. They highlighted that the optimal solution for medium-to-large rural healthcare facilities is a hybrid system coupling a renewable energy source with efficient batteries and a diesel generator to minimize the cost of coping with the intermittency of renewable energy sources. Orosz et al.^30^ examined technical and economic options for electricity, heating, and cooling in health and education applications in rural SSA. They noted the significant benefits and cost savings of solutions based on photovoltaics hybridized with liquefied petroleum gas/propane and micro-concentrating solar power tri-generation, compared with conventional diesel and LPG/propane-based heating and cooling. The World Resource Institute’s Energy Access Explorer provides information on health center locations, electricity demand, and renewable energy potential for Kenya, Uganda, and Tanzania.^31^ The HOMER Powering Health Tool^32^ (USAID, ESMAP, WeCareSolar) is a free online model to create initial designs of electric power systems for healthcare facilities that have no other power supply (i.e., diesel generator) or have grid electricity available for a predictable period of hours each day.

Our study builds on such country-specific research and is the first of its kind to carry out a continental-level assessment of the electrification access status for healthcare facilities (the African electricity access health facility geodatabase: Data and compilation procedure and population clusters and healthcare facility catchment areas), including estimates of the travel time to each of the facilities with and without electricity (population clusters and healthcare facility catchment areas). Our analysis also estimates (1) the energy requirements for powering the health centers and the optimal size of the PV and battery system (estimation of health facility energy demand) and (2) the associated costs (assessment of electrification costs). The results are presented at continental, regional, and national levels.

Until recently, the main electricity supply options considered in rural areas of SSA were the extension of the national grid or the use of standalone diesel generators. This analysis explores the potential of electrifying healthcare facilities in rural areas using PV mini-grids (including batteries). A multi-criteria algorithm is used to identify healthcare facilities with a high probability of having no electricity access (referred to as no electricity access [NEA] healthcare facilities). To do so, we collected, harmonized, and aggregated a range of open-source datasets (see experimental procedures).

**Figure 3:**
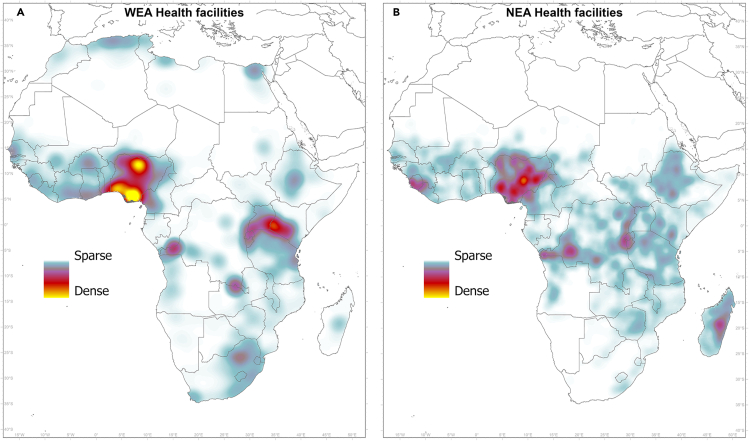
Heatmaps of healthcare facilities according to their electricity access status (A and B) For (A) facilities with detected electricity access (WEA) and (B) facilities with no detected access to electricity (NEA).

Figure 3 shows heatmaps of healthcare facilities according to their electricity access status. The total number of mapped facilities is 122,899 for the whole African continent, categorized as (1) those with no access to electricity (NEA) (in total, 56,801) and (2) those in locations with detected electricity access (WEA) (in total, 66,098). Of the NEA healthcare facilities, 96% are primary health posts (offering very basic services), and a small number are primary (3%) and secondary (1%) hospitals (see estimation of health facility energy demand and Table 2). The heatmap in Figure 3A highlights areas with a higher concentration of facilities without electricity access (yellow); an example of a dense concentration is in Nigeria, with more than 13,000 NEA facilities.

## Results

Following the methodology described in experimental procedures, the study identified 56,801 health centers in SSA that fall in the NEA category. For each NEA facility, we assessed the costs of providing electricity with PV decentralized systems and identified the population served under several travel time assumptions. Table 1 summarizes the key data aggregated at the country level, while the following sections summarize the results at various geographical levels.

**Table 1:** Electrification of healthcare facilities by PV off-grid systems: total investment costs, average LCOE and share of population

Country		Population					Healthcare facilities			Costs		
		Total	Pop. 20 min walking to all facilities	Pop. 20 min walking time to WEA	Pop. 60 min travel time to all facilities	Pop. 60 min travel time to WEA	Total number of facilities	Number of NEA facilities	Total PV capacity	Average LCOE	Total upfront costs	Total NEA upfront costs
		[million]	[%]	[%]	[%]	[%]	–	–	[kWp]	[EUR/kWh]	[EUR million]	[EUR million]
AGO	Angola	25.0	53	51	94	92	1,707	861	4,474	0.37	16	6.3
BDI	Burundi	11.1	70	50	100	100	775	329	2,876	0.44	10	3.3
BEN	Benin	10.9	64	63	100	99	970	67	2,464	0.39	9	0.5
BFA	Burkina Faso	18.1	63	58	96	95	1,863	519	4,287	0.36	16	3.7
BWA	Botswana	2.3	65	64	99	98	671	224	1,665	0.41	6	1.7
CAF	Central African Rep.	4.9	59	32	97	56	820	715	2,600	0.41	10	6.8
CIV	Cote d'Ivoire	22.7	62	58	99	99	2,004	383	6,115	0.39	22	2.9
CMR	Cameroon	23.3	81	74	99	96	3,540	1,724	10,504	0.42	38	15.8
COD	Dem. Rep. Congo	77.3	62	38	98	67	14,746	9,790	39,658	0.42	148	100.7
COG	Congo	4.6	67	61	95	90	355	167	1,050	0.43	4	1.5
COM	Comoros	0.8	62	62	100	100	71	0	83	0.22	0	0
DJI	Djibouti	0.9	44	44	91	87	67	30	764	0.31	3	0.2
ERI	Eritrea	5.3	60	56	79	75	276	173	646	0.34	2	1.1
ETH	Ethiopia	99.2	48	30	95	89	5,399	3,534	12,209	0.40	44	26.3
GAB	Gabon	1.7	59	54	99	93	560	397	1,571	0.43	6	3.3
GHA	Ghana	27.4	58	54	100	99	2,092	640	5,478	0.39	19	4.8
GIN	Guinea	12.6	58	42	99	94	1,648	1,007	3,565	0.37	13	6.9
GMB	Gambia	2.0	59	56	100	100	123	26	251	0.34	1	0.2
GNB	Guinea-Bissau	1.8	28	27	81	80	14	2	186	0.37	1	0.1
GNQ	Equatorial Guinea	0.8	42	29	99	99	50	18	292	0.45	1	0.3
KEN	Kenya	46.0	54	49	96	90	6,262	1,712	15,148	0.39	54	12.5
LBR	Liberia	4.5	63	48	97	91	909	537	2,230	0.42	8	4.4
LSO	Lesotho	2.1	34	30	100	100	184	42	820	0.49	3	0.4
MDG	Madagascar	24.0	30	15	78	45	4,175	3,723	9,187	0.39	33	28.2
MLI	Mali	17.6	40	34	93	78	1,845	1,278	3,752	0.35	14	8.4
MOZ	Mozambique	27.8	51	40	92	82	1,643	852	10,702	0.41	38	20.9
MRT	Mauritania	4.0	49	47	80	74	712	348	1,146	0.31	4	2.1
MUS	Mauritius	1.3	84	84	100	100	176	0	426	0.36	1	0
MWI	Malawi	17.3	20	15	100	98	700	353	2,401	0.41	9	2.9
NAM	Namibia	2.5	39	38	91	90	393	69	879	0.33	3	0.4
NER	Niger	19.9	52	40	89	76	3,018	1,943	4,834	0.31	18	11.2
NGA	Nigeria	182.1	80	71	99	98	36,428	13,060	93,701	0.40	339	108.9
RWA	Rwanda	11.6	50	32	100	100	607	276	2,146	0.43	8	2.5
SDN	Sudan	40.2	27	26	70	69	491	134	2,022	0.30	8	3.2
SEN	Senegal	15.1	72	69	99	98	1,538	464	2,947	0.33	11	3.0
SLE	Sierra Leone	6.4	69	43	100	87	1,808	1,327	4,084	0.41	14	10.3
SOM	Somalia	10.8	60	43	94	71	865	618	1,628	0.30	6	3.8
SSD	South Sudan	12.3	58	34	88	67	1,779	1,355	4,188	0.40	15	10.8
STP	Sao Tome Principe	0.2	67	67	97	97	54		249	0.39	1	
SWZ	Swaziland	1.3	27	26	100	99	139	22	447	0.56	2	0.2
TCD	Chad	14.0	64	50	90	72	1,570	1,258	3,785	0.35	14	10.0
TGO	Togo	7.3	50	47	99	97	363	141	1,571	0.40	6	1.3
TZA	Tanzania	53.4	57	39	96	90	7,547	3,840	17,363	0.39	62	28.5
UGA	Uganda	39.1	47	45	99	99	4,404	533	10,000	0.40	36	3.9
ZAF	South Africa	54.5	52	51	100	100	4,713	377	17,191	0.49	66	3.8
ZMB	Zambia	16.2	31	29	95	86	1,388	893	3,539	0.37	13	7.0
ZWE	Zimbabwe	15.6	27	25	87	67	1,437	1,040	4,275	0.43	15	8.7
	Total/average	999.8	54	70	91	88	122,899	56,801	321,399	0.39	1,170	484

The number of population represents the amount of population living in areas within the indicated travel time (60 min optimal travel time or 20 min walking). The total investment costs (for components, engineering, and soft costs) are calculated aggregating the total cost of decentralized energy options, taking into account the optimized size of the system for each health facility with its specific load consumption and the economy of scales (lower upfront cost for larger systems). The LCOE is calculated as an average of the LCOE values per country, taking only the NEA facilities serviced by PV.

### Estimation of population served

**Figure 4:**
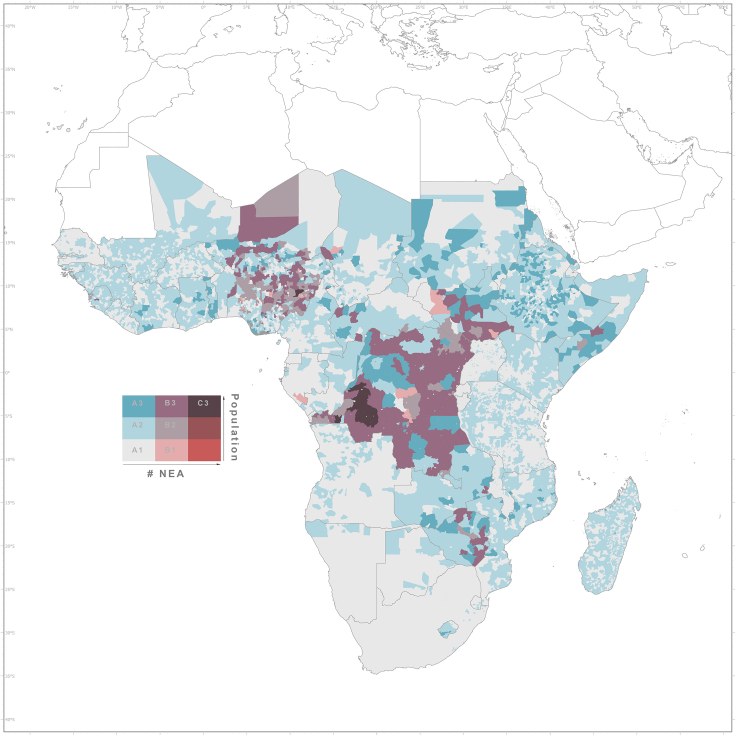
Bivariate representation at administrative level 3 of the percentage of NEA healthcare facilities and the percentage of population without access to electricity The percentage of NEA healthcare facilities ranges from light blue hue (low values) to dark (high values), while for population with electricity access ranges from light red hue (low values) to dark (high values).

The bivariate map (Figure 4) provides a geospatial overview of the most critical level 3 administrative units in terms of lack of electricity access for the population and for health facilities. The percentage of NEA healthcare facilities ranges from low (left in the legend) to high (right) and the percentage of the population with electricity access from low (bottom in the legend) to high (top in the legend). Gray light colors (A1 in the legend) indicate areas with high electricity access rates for both the population and healthcare facilities. Dark cyan color shades (A3 in the legend) represent areas with high electrification rates of facilities but low electricity access rates for the population. The darkest mahogany shading (B3, C3) defines areas with low rates of electricity access for both the population and healthcare facilities, for example, predominantly in regions in Central Africa. Figure 4 also identifies areas with an asymmetric relationship between the two measures of electricity access. For instance, areas with a high level of electricity access to healthcare facilities but where the general population still has a low access rate (dark blue).

Unsurprisingly, a review of the literature on healthcare service location found that the relationship between the proximity to healthcare facilities and health outcomes is significant.^33^ Therefore, to estimate the potential benefits of electrifying healthcare facilities, the analysis focused on quantifying travel time to the nearest facility, either currently electrified or not. Two options are considered: travel by the fastest available transport mode (optimal travel time) and walking time. The latter is particularly relevant to the rural locations and small primary health posts that make up the majority of NEA healthcare facilities in this study.

**Figure 5:**
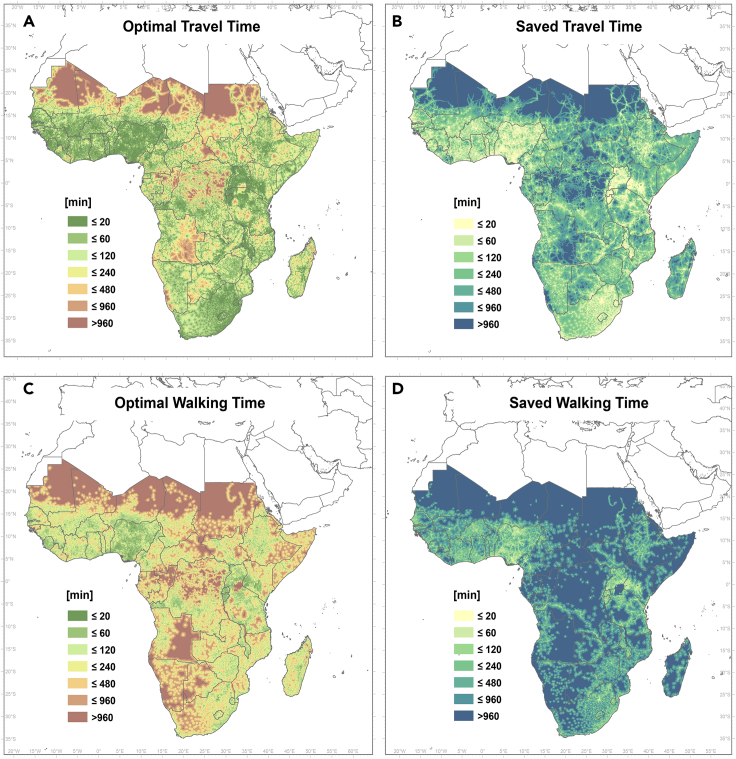
Travel times to healthcare facilities (A and B) (A) Optimal travel time (min) to the most accessible healthcare facility (to all facilities, including NEA) compared with (B) additional time required when traveling to only a facility with electricity (WEA). (C and D) (C) Optimal walking time (min) to the most accessible healthcare facility (all including NEA) compared with (D) the additional time required when walking to a facility with electricity (WEA).

Figure 5A maps the travel time to the most accessible healthcare facility at each location (by any means of transport) and Figure 5B shows the additional travel time needed to get to the most accessible health facility that already has access to electricity. The underlying accessibility estimation methodology is described in the study conducted by Weiss et al.^34^ Note that this analysis only focuses on facilities that can be reached most rapidly and ignores complexities such as individuals choosing to go to more distant facilities because of specific preferences (e.g., public/private hospitals). Also, there is no systematic database of private healthcare facilities in SSA. Several hotspot regions are evident (e.g., the Democratic Republic of the Congo, Madagascar, and Chad), where significantly longer journeys are required for people needing access to healthcare facilities with electricity. Figures 5C and 5D show the same comparison for travel times by foot (a prevalent travel mode in rural areas). Here, the difference is even larger in some areas—a significant discrepancy is observed in almost all countries from Eastern and Central Africa, especially in Somalia, Ethiopia, the Democratic Republic of the Congo, the Central African Republic, Chad, and Madagascar. Concerning the differences in healthcare accessibility between countries in Western and Central Africa, the disparity is largely due to population density and, to a lesser extent, the completeness of the healthcare facility dataset for each country. For example, Nigeria has a population of over 200 million, whereas DRC (second largest population in Africa and 2.5 times larger in area) has about 90 million. Nigeria also has more than 3 times the number of healthcare facilities. For these reasons, the accessibility map shows that people in West Africa are closer to healthcare facilities.

**Figure 6:**
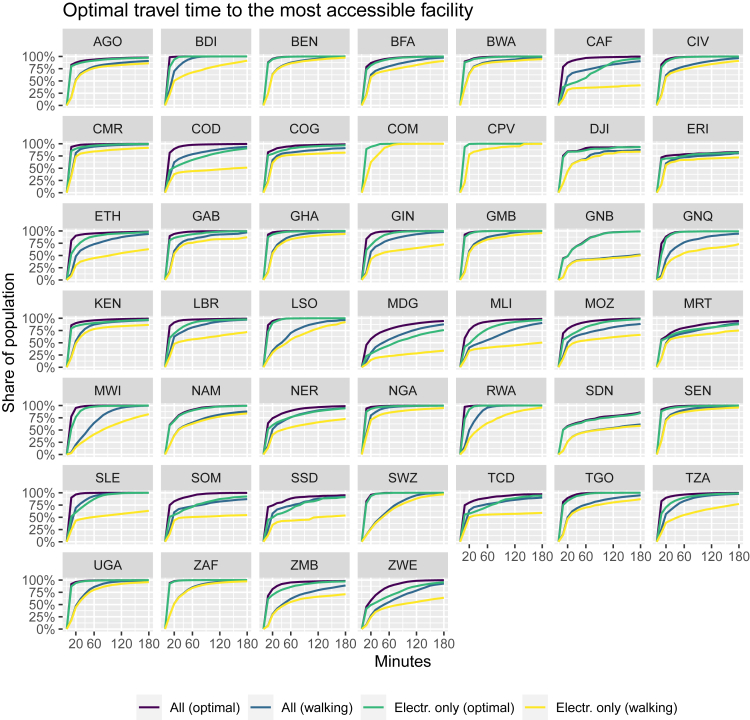
Comparison of the accumulative population at optimal travel time (min) to most accessible facility To all facilities (including NEA, violet line), to the nearest electrified health facility (WEA, green). For walking time to all most accessible facilities (including NEA, blue), and to the nearest electrified health facility (WEA, yellow)

In terms of the population affected, Figure 6 shows cumulative curves of the share of population in each country with a given travel time to the most accessible healthcare facility. These curves illustrate the accessibility inequality between countries for all health centers (purple lines) compared with the health centers with access to electricity only (green lines). Figure 6 also highlights the pronounced differences between walking time distance to any facility (blue lines) and to only electrified healthcare facilities (yellow lines). For instance, a greater percentage of the population of Nigeria (NGA) can rapidly access healthcare facilities than in Equatorial Guinea (GNQ). Disparities between countries are larger for walking travel times. In the Democratic Republic of the Congo, only about 50% of the population can reach an electrified health facility in less than 60 min by foot. Note that the 60 min threshold for traveling on foot generally approximates to the 5 km buffer found to be meaningful for determining the propensity for care seeking.^35^

At the continental level, only 4% of the sub-Saharan African population lives within >1 h of travel time from a health facility, but 10% live within >1 h travel time from an electrified health center. Considering travel by foot, 46% of the SSA population lives within 20 min or more of walking time from a health facility, while 54% from an electrified health center. Limitations on access to electrified facilities is higher in some countries: for instance, in Madagascar, 55% of the population live more than 1 h from the nearest electrified facility and 85% with more than 20 min of walking time. In contrast, in Malawi, only 2% are at 60 min or more, although the value for >20 min walking time is similar to that of Madagascar (70%). The Central African Republic (∼44% optimal travel time), the Democratic Republic of the Congo (∼32%), and South Sudan (26%) also had high percentages of the population living further than 60 min away from electrified facilities. Figure 6 and Table 1 report detailed country-level accessibility statistics.

### Estimation of the electrification costs for the NEA healthcare facilities

**Figure 7:**
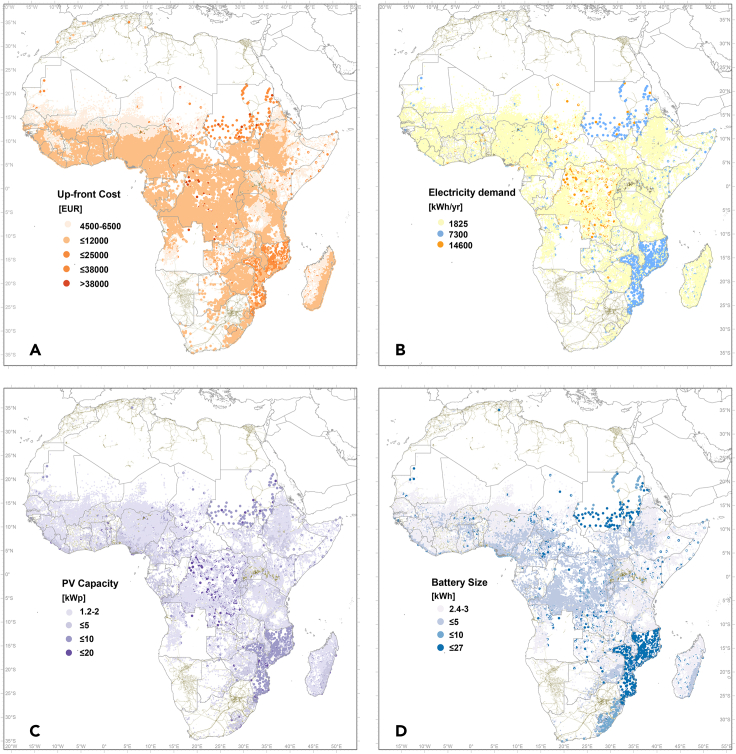
Distribution maps for NEA healthcare facilities (A) Upfront cost of NEA healthcare facilities (EUR). (B) Estimated annual electricity demand per health center (kWh/year) (C) Optimized PV capacity requirement (kW_p__)_. (D) Optimized battery storage requirement (kWh). Note that the high electricity demand for healthcare facilities in Sudan and Mozambique reflects the assigned classification; field data would be needed to fully harmonize this across the continent.

**Figure 8:**
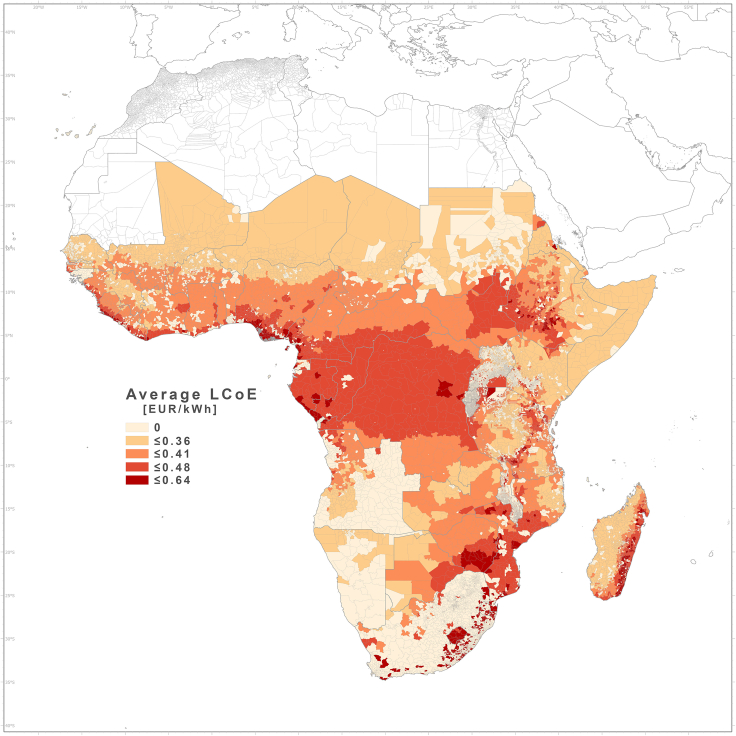
LCOE (EUR/kWh) for PV systems installed in NEA health centers LCOE of decentralized PV systems (including storage) for the NEA healthcare facilities in Africa average at administrative level 3.

**Figure 9:**
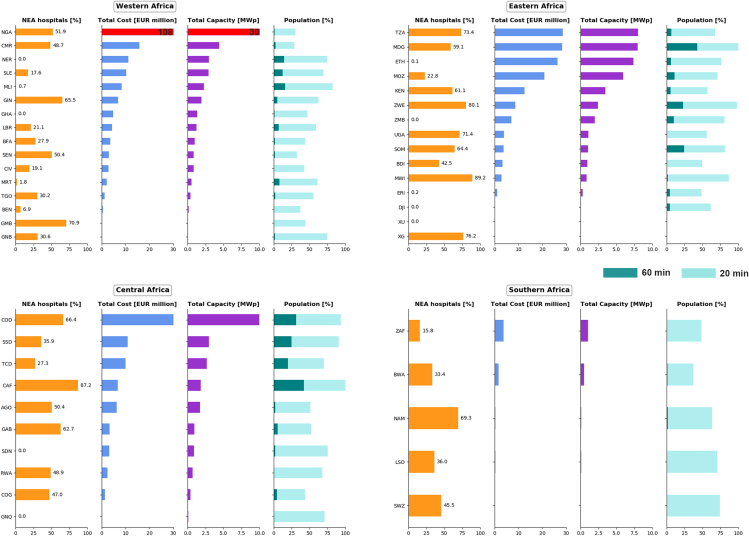
Percentage of NEA healthcare facilities per country (orange bars, first column) Total PV capacity (MW_p_) per country estimated to be installed in the NEA healthcare facilities (purple bars). Total costs (million EUR) of installing PV systems to NEA healthcare facilities (blue bars). The share of population with more than 60 min travel time to an electrified health facility (dark green bars) and the percentage of population with more than 20 min walking time to an electrified health center (cyan bars).

Next, we estimated the optimized PV capacity, the optimized battery capacity, and the associated costs for each of the healthcare facilities (Figure 7; Table 1), and then aggregated them to estimate the total capacity and costs at the national level (Figures 8 and 9). Details of the underlying data, calculations, and assumptions are found in experimental procedures. Figure 7A shows the estimated PV upfront cost; Figure 7B shows the estimated annual electricity demand based on the levels of NEA healthcare facilities. Figures 7C and 7D show the calculated PV system power capacity requirement [kW_p_] and battery storage capacity requirement [kWh], respectively. These values are location dependent. For primary health posts with the same electricity demand (1,825 kWh/year), the optimized PV system size ranges from 1.2 to 2.8 kW_p_ and battery size from 2.4 to 7 kWh. The two extremes correspond to locations on the coast of Somalia, which has high solar radiation and lower seasonality, and on the South African east coast, which needs a larger PV array and battery size due to lower irradiation and distinct winter/summer seasons. The estimated PV upfront cost (including hardware, engineering, and soft costs) varies correspondingly from EUR 4,500 to EUR 11,000.

The levelized cost of electricity (LCOE) was computed for each of the healthcare facilities, with values ranging from 0.25 EUR/kWh in Somalia to 0.62 EUR/kWh in central Nigeria. Figure 8 shows the average LCOE values per region. Large geographical differences can be observed, generally depending on the local climatic conditions^36^ and degree of seasonality.

To estimate the total generation capacity per country required to power all the rural health facilities, the PV capacities of both the WEA and NEA facilities were aggregated (Figure 9). In a similar way, the overall costs were estimated at the national level (Figure 9, blue bars) for rural healthcare facilities, reaching a total upfront investment cost for all SSA of EUR 1,170 million. If only the NEA facilities are considered, the sum is EUR 484 million. The cost of providing universal access to healthcare facilities with electricity in each country will clearly depend on the percentage of healthcare facilities that already have access to electricity. For example, in Kenya and Tanzania, the total PV capacity required to cover all health centers is in the same range (15 MW_p_ and 17 MW_p_, respectively), but the costs of electrification for all NEA facilities are much higher in the case of Tanzania (EUR 28.5 million) than in Kenya (EUR 12.5 million) because in Tanzania, 50% of health centers do not have access to electricity compared with 26% in Kenya.

**Figure 10:**
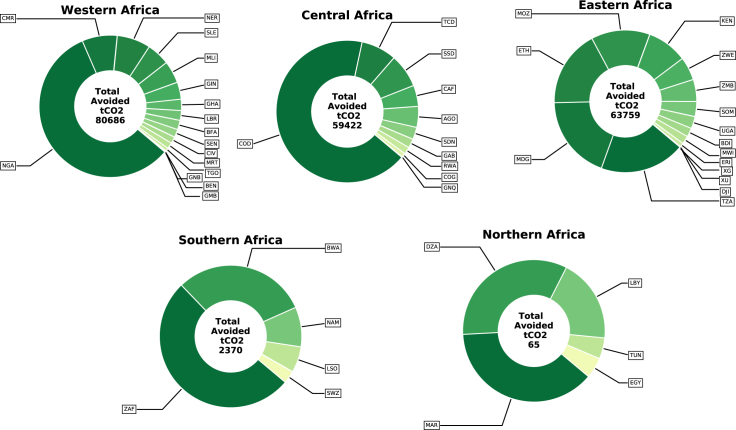
Breakdown of avoided GHG emissions (tCO_2eq_) estimated per NEA health center and aggregated per country in each African region

In addition, the avoided greenhouse gas (GHG) emissions were calculated by computing the emissions of a standalone diesel generator supplying the same electricity demand over the lifetime of the optimized PV system per health center (see experimental procedures). Figure 10 shows the estimations of the total avoided GHG emissions per country when powering all the rural health facilities with renewables. In addition, the total GHG emissions avoided amounted to 206 kt of CO_2_ over a system lifetime of 20 years.

**Figure 11:**
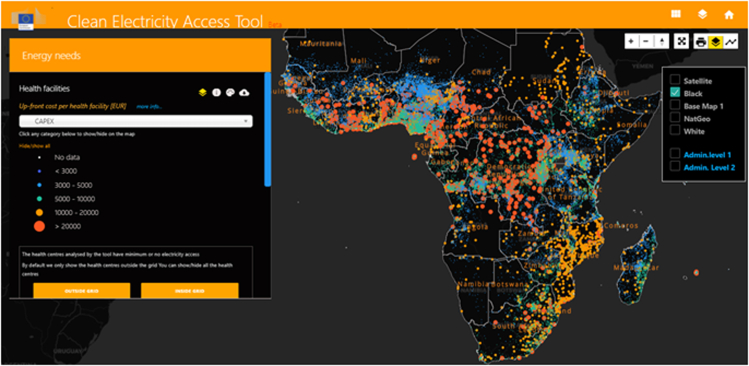
Clean energy access tool, including healthcare facilities analysis Open-source web tool developed by the European Commission-JRC (http://d6-dev-africap.jrc.it/energy_tool).

**Figure 12:**
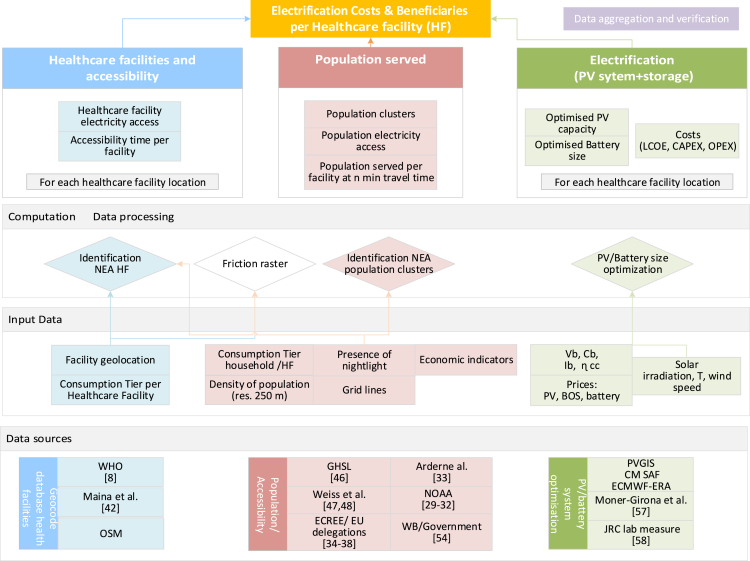
Simplified methodology framework showing the data sources and the various stages in the generation and validation of the data: inputs, processing, and outputs

An additional aspect of sustainability is the end-of-life management of PV-battery mini-systems, as addressed in several studies.^37^^,^^38^ The volume of PV modules for NEA electrification is projected to be approximately 5 MW, with a useful life of 25 years. This is less than 0.5% of the 2020 PV market volume in Africa.^22^ Analysis of recycling and e-waste policies is beyond the scope of this paper, but it is expected that the products used in rural electrification will be able to take advantage of the national schemes needed to address the overall market.

Table 1 summarizes the total investment costs, average LCOE, and the population that can reach a potentially new-electrified healthcare facility within 1 h motorized travel time and 20 min by foot for each country.

To conclude, the datasets created in this work are being used to provide free online information via the Clean Energy Access (CEA) Tool (accessible at http://d6-dev-africap.jrc.it/energy_tool). This has been developed for visualizing and analyzing information on electricity access in Africa and the overall clean energy outlook. The tool (Figure 11) has a specific focus on improving the general healthcare in areas of rural Africa with minimal or no access to electricity. It allows the visualization and analysis of the EHFDB healthcare facilities singularly or for user-defined areas. The results can be summarized at the national and/or subnational levels and then downloaded. The EHFDB database is also available for download from https://data.jrc.ec.europa.eu/collection/id-0076).

## Discussion

Our results shed new light on the potential of decentralized energy systems to offer a reliable, quick, and cost-effective way to increase access to electricity in rural healthcare facilities in SSA. This study identified 56,801 NEA health centers in SSA and analyzed the costs and benefits of powering these by decentralized PV systems.

We estimated that 281 million people would benefit from reduced travel time to a health facility with electricity access if all NEA facilities were provided with PV systems (reaching an average of 50 min). The impact is even more pronounced when considering only walking as a means of traveling, in which case, 298 million people would reduce travel time (by an average of 6 h) if all current NEA facilities were provided with electricity. This highlights a particularly urgent need to power those healthcare facilities which serve populations with above-average journey times to health posts and hospitals with electricity. The analysis reported here provides a means to easily identify such priority areas.

There are no significant resource or technical barriers to using solar photovoltaic-based systems to provide electricity for rural healthcare facilities. The PV/battery system size optimization was computed for 122,899 healthcare facilities, assuming a level of electricity demand depending on the type of healthcare facility (according to the WHO electricity demand tiers) and a full coverage of over 95% of days per year (286 GWh/year in total). Under the chosen assumptions, an annual energy demand of 121 GWh would be needed to cover the needs of the sub-Sahara African NEA rural health centers. Up to 2020, Africa, the continent with the richest solar resources in the world, had installed 11 GW_p_ of solar PV, about 1% of the global total.^22^^,^^39^ According to the IEA Africa projections,^5^ PV deployment should grow to almost 15 GW_p_ a year and is projected to reach 320 GW_p_ in 2040. The results of this study estimated the total current demand for NEA facilities will be satisfied by 133 MW_p_ of solar PV (with 220 MWh of battery storage), which is small relative to the above projects but would potentially have an enormous societal impact. In addition, electrifying NEA using PV compared with non-renewable sources would avoid 206 t CO_2_ in GHG emissions.^40^

The LCOE of the PV systems (including battery storage) installed in the NEA averages to 0.4 EUR/kWh, with a range from 0.26 to 0.64 EUR/kWh (Table 3). These values are lower than those reported up to now for mini-grid systems in Africa. However, the cost is decreasing, and the intention is to show what could be affordable with economies of scale, standardized products, more experienced local suppliers, and efficient administrative procedures. It is important to note that when the LCOE is calculated without design, installation, or permitting costs—for instance, for comparisons with other generation renewable or fossil technologies—the LCOE values vary between 0.16 and 0.49 EUR/kWh.

The total upfront investment cost of powering the existing SSA NEAs by PV systems (including battery storage and soft costs) totals to EUR 484 million. This sum is relatively minor compared with the EUR 15.6 billion financial flows in 2016, supporting clean and renewable energy in developing countries,^5^ as well as the latest IEA figures for achieving universal electricity supply in Africa of around EUR 92 billion a year through 2040. The funds we envisage to support PV expansion for NEA include those aimed at achieving SDG3 on good health and well-being, where electrification would be part of an overall package of measures. This further increases the policy relevance of our bottom-up assessment. Overall, the analysis presented in this paper and the associated open-source web tool (http://d6-dev-africap.jrc.it/energy_tool) can support a better integration of energy and health policy by identifying the areas/countries where the investments are most needed. Already, some development agencies are mobilizing investments in the direction suggested by our paper.^32^^,^^41^^,^^42^

Despite the large amount of data collection, computing, and analysis involved in the development of the study, limitations remain. The data-intensiveness of the analysis implies growing uncertainty over the reliability of the database, as some sources such as the existing grid infrastructure and facilities locations and characteristics might be outdated or incomplete. For example, there are limited data available on electricity grid lines in a number of sub-Saharan African countries. Another limitation of the approach is the use of night-time lights to estimate electrification as, for example, it could miss facilities with standalone electricity access closed in the night or/and not having outdoor lights. The methodology does not address the existing unreliability of the grid electricity, which is also a key factor to consider in terms of the quality and continuity of service a health facility can provide. In the analysis, we rely on the Global Human Settlement Layer (GHSL) gridded population product, but adoption of different population products might lead to slightly different results, as highlighted in study conducted by SDSN and TReNDS.^43^ Moreover, gridded population products are based on statistical downscaling of census data and thus systematically exclude invisible populations, as reported in the study conducted by Carr-Hill et al.^44^^,^^45^ Although beyond the scope of our paper (which focuses on meeting the current needs of existing facilities), future research could use the results of modeling studies projecting future facility needs based on population growth and the accessibility and/or patient beds target-based in our study^33^ to also estimate the potential future demand arising from adding new facilities. In addition, the accessibility analysis excludes individual preferences that may push individuals to seek healthcare at a facility that is not the most accessible one. Finally, the classification of facilities for each country might have limitations, as very different national systems have been manually homogenized into generally valid healthcare tiers.

Validation of the applied model was completed by partially available health facility data (e.g., Gambia) and visual interpretation of satellite images. However, in specific countries (such as Zambia and Rwanda), the lack of complete information on medium voltage and low voltage grid lines leads to larger numbers of NEA centers than the publicly available statistics (39% instead of 20% and 30% instead of 18%, respectively). Several institutions (including the WHO) are currently performing a global assessment of electricity in healthcare facilities with the aim of closing the existing knowledge and lack of information,^2^ and the outcomes can support the validation of the methodology used here and increase the accuracy of the analysis.

Nonetheless, our methodology is a first approximation to quantify and analyze the current situation for all of SSA and can provide a basis for further studies that can take advantage of complementary field data collection to create a more comprehensive database of energy access levels for all social infrastructure, not just health facilities. To address this need, data collection should aim to offer a multidimensional picture of the availability and reliability of existing electricity services, either off-grid or on-grid. This study also highlights the importance of the aggregation of multiple data sources, both from national or regional surveys and field data collection that promotes better understanding of the subject. Representing information with a geospatial dimension can further help outline the multifaceted picture of energy access in health centers in Africa. Also, the results provide an opportunity for future studies to address factors such as a more detailed representation of the health center load profiles, including seasonal variability, future increases in electricity demand with economic and demographic growth, and the use of PV systems for grid-connected health centers to ensure power reliability.

Our results can be considered as a promising aspect for planning the electrification of health facilities in rural SSA and are potentially beneficial for policy makers, researchers, consultants, and other stakeholders involved in electrification planning and healthcare improvement. The level of granularity, covering community, national, and regional levels, is particularly relevant to the prioritization in the allocation of limited governmental funding, highlighting regions where electrification is most needed and likely to have the greatest impact on health services for rural populations. Effective strategies for financing electrification of healthcare are critical and remain a challenge in SSA.^46^ Therefore, when planning at the national level, it is of critical importance to take into account how programs are designed and what priorities are applied by national and/or local authorities. Establishing evidence-based and multisectoral strategies tailored to each country-specific context remains imperative. Given the complex and multifaceted nature of sustainable energy access, composite indicators can help attract investment in decentralized electricity generation. An example of this is the PV Decentralized Energy Investments (PV-DEI) index,^47^^,^^48^ which covers the environmental, social, political, and financial aspects with over 50 individual indicators. High scores in the social dimension imply that the impacts of investing in decentralized PV are likely to significantly improve various social outcomes. The methodology introduced in this study could be extended to assess how solar energy for health facilities and other social infrastructure can be developed as part of an integrated energy hub for off-grid rural communities.

## Experimental procedures

### Resource availability

#### Lead contact

Further information and requests for resources should be directed to and will be fulfilled by the lead contact, Magda Moner-Girona (magda.moner@ec.europa.eu).

#### Materials availability

All unique materials generated in this study are available from the lead contact without restriction, Magda Moner-Girona (magda.moner@ec.europa.eu)

### The African electricity access health facility geodatabase: Data and compilation procedure

Information on electricity access for healthcare facilities is scarce, not collected systematically, and limited to specific projects or regional aggregations. As a result, we developed an electricity access health facility database (EHFDB) in Africa. Tthe EHFDB database can be downloaded from https://data.jrc.ec.europa.eu/collection/id-0076 for this study using various source data. We accounted for the lack of available data related to the status of electricity access of African health centers by extracting information from the available satellite remote sensing archives and the spatial extent of the existing electricity grid.^49^, ^50^, ^51^, ^52^, ^53^ For this study, a new electricity grid layer was compiled using multiple sources regarding the existing transmission and distribution network. These include the Open Street Map, the World Bank datasets, Arderne et al.,^54^ the Economic Community of West African States Observatory for Renewable Energy and Energy Efficiency,^55^ as well as rural electrification agencies and EU delegations in Africa (Burkina Faso,^56^ Kenya,^57^ Tanzania^58^^,^^59^).

**Figure 13:**
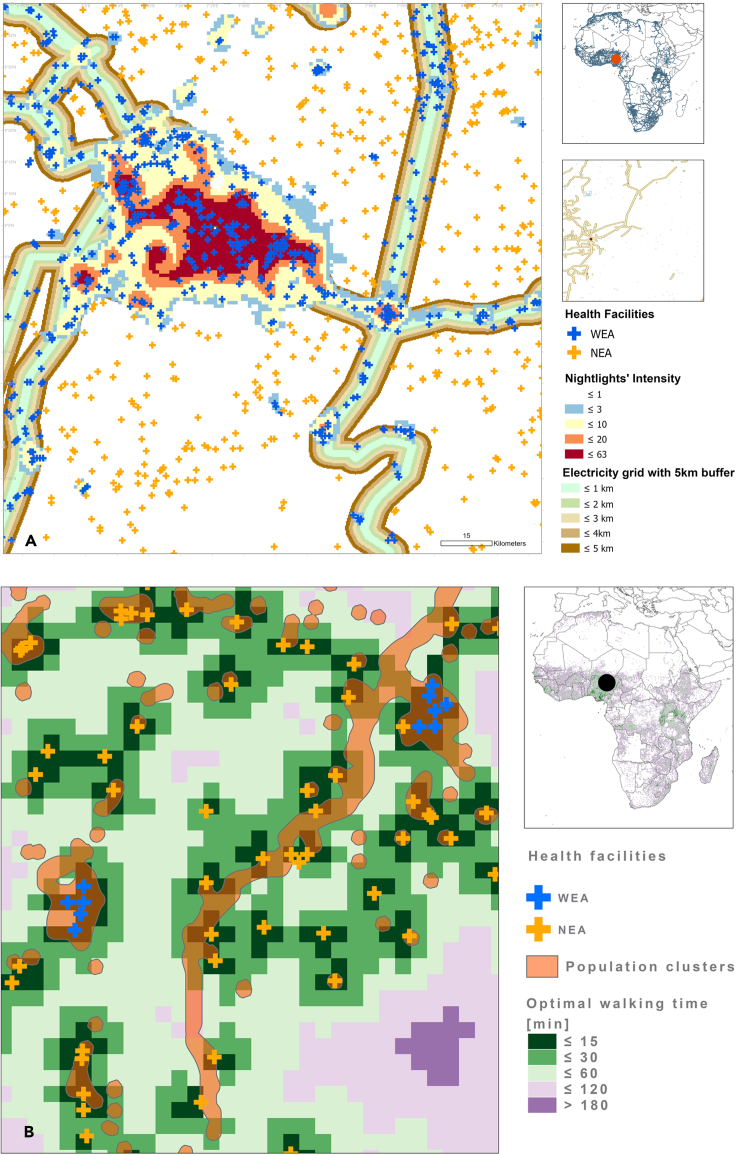
Zoom-in maps for an area with healthcare facilities (A) Zoom-in map of the healthcare facilities classified as (A) with electricity access (WEA) in blue crosses and no electricity access (NEA) in orange crosses. The nightlights’ intensity background layer (buffer from yellow [low intensity] to red [highest intensity] )and the electricity grid 5 km buffer (in shaded brown). (B) Zoom-in area with background layer, the optimal travel time (by foot in minutes) to healthcare facilities with access to electricity: the shortest walking time (<15 min) is represented in green to the longest time in violet. The map includes clusters of population (light orange polygons), WEA and NEA healthcare facilities. The maps were generated using the following data, which was collected and processed by the authors: GHS population grid; GHS-POP 16 data, produced and made publicly available by the European Commission – JRC (https://ghsl.jrc.ec.europa.eu/data.php); and night-time lights Version 4 DMSP-OLS 17, produced and made publicly available by NOAA's National Geophysical Data Centre (VIIRS DNB) (https://ngdc.noaa.gov/eog/dmsp/downloadV4composites.html).

A buffer zone of 5 km was created around the geo-located electrification infrastructure to identify areas likely to be able to connect to the electricity grid (https://data.jrc.ec.europa.eu/dataset/624c6e71-3b9c-4f48-8c67-645911798d41). In parallel, we utilized night-time lights data captured by a sensor aboard the NASA-National Oceanic and Atmospheric Administration NPP satellite published in 2019 with a 450 m^2^ resolution. The nightlight’s intensity layer was used to define areas with the presence of night-time lights.^50^, ^51^, ^52^, ^53^^,^^60^, ^61^, ^62^ By combining the buffered electricity grid and the nightlight layer mask, we created a proxy layer of electrification coverage (Figure 13). Night light with an intensity of less than 0.5 was treated as noise and excluded from our analysis.

The resulting EHFDB incorporates (1) the geographic locations of health centers acquired from the healthcare facilities spatial database published by Maina J. et al.,^45^^,^^59^ combined with open-source data, such as OpenStreetMap (OSM; https://www.openstreetmap.org/) and Google Maps (https://www.google.com/maps/), (2) a geostatistical probabilistic layer on electricity access for healthcare facilities,^61^^,^^62^ and (3) the estimated power requirements, the optimized PV and battery size to meet these requirements, and the costs of the systems to be installed, calculated as described in the next sections.

### Population clusters and healthcare facility catchment areas

Since an accurate and high-resolution geographical distribution of the population is essential to determine the population without access to electricity, our spatial analysis used the integrated continental dataset of population distribution (published in 2019) provided by the GHSL framework.^64^, ^65^, ^66^, ^67^ The GHSL builds on past research and relies on processing 40 years of Landsat imagery for mapping the global built-up areas from 1975 to 2015.^64^^,^^65^ The population grid datasets (GHS-POP) were derived from the GHSL building density and population census data and were originally developed to allocate census population data in built-up areas.

Population clusters were generated using the latest GHSL continental population layer at a 250 m resolution.^67^ The ArcGIS “regions creation” functionality was used as a first step to identify the population clusters instead of people per single pixel (cell). This allowed us to use the connectivity option with 8 neighboring cells, whereby adjacent cells could become a part of the same cluster. Once the first clusters were defined, a second step was used to connect the clusters within a 250 m distance or one pixel. Populations were allocated to each unique cluster by aggregating the population for each pixel contained in the delineated cluster. A geospatial mask layer combining the proxy layer (electricity grid with its 5 km buffer and the night light mask where lights are present) was used to delineate population clusters with a probability of having access or those without access to electricity. For practical purposes, this provides a way to estimate electricity access and, therefore, to estimate the population in areas without electricity access (see Figures 13A and 4). Figure 13B shows an example of an area with the defined population clusters, where the NEA healthcare facilities are represented as orange crosses and the WEA facilities as blue crosses.

#### Healthcare facility catchment areas

The number of potential beneficiaries for each health facility is obtained from estimates of the population within a certain travel time. Continent-wide travel time maps were generated for both on-foot travel and motorized travel using an established accessibility estimation approach.^34^ This approach is predicated on applying a least-cost algorithm to a set of geo-located points in combination with a friction surface containing estimates of the time it takes to traverse each pixel within a global grid with a spatial resolution of 30-arc-s (approximately 1 km at the equator).^33^^,^^34^^,^^68^, ^69^, ^70^, ^71^ The resulting travel time maps were then used to cleave the population surface into pixels, and thus the population, beyond a set of minute thresholds from the nearest facility. We also generated facility catchments, defined as all pixels that have the shortest travel time to a given healthcare facility. We next calculated the zonal sum for each facility catchment from the population layers. Lastly, we intersected the facility points with the catchment layer to account for instances when multiple facilities were located within the same 30 arc-s pixel, as defined by the resolution of the travel time analysis. In doing so, each facility was associated with a potential patient population, even if that population was shared among several co-located facilities. The result of this analysis consists of the original table of facilities, amended with additional columns containing the population of the associated facility catchment. Note, however, that attributing populations to facilities in this manner ignores individual decisions to seek care from more distant facilities or none at all.^72^

### Estimation of health facility energy demand

The WHO, along with the World Bank, have developed a multi-tier measurement of electricity supply in primary and secondary healthcare facilities,^6^^,^^9^^,^^73^ along with typical functional profiles divided in several tiers^74^. In this analysis, we link the electricity demand of these tiers to 4 types of healthcare facilities (Table 2) (at the primary level, these vary between countries in their definitions, specialization, population served, services provided, infrastructure, and staffing. Health centers, medical centers, polyclinics, health posts, dispensaries, clinics, health huts, health units, etc. may have similar functions but may equally represent different levels of service provision between countries). The appropriate healthcare electricity demand tiers have been defined following the WHO levels of health services of primary, first referral, second referral, and tertiary referral^8^ levels. Given that there is no universal standardized definition of health facility types, making cross-country comparisons is difficult, particularly at the primary level, as these vary between countries in their definitions.^63^ Each health facility was checked, and a manual labeling was applied to classify them in the 4 categories, using database queries based on the provided names of each facility or the type of the facility if available. This was achieved by extracting unique facility-type names, assigning the tier value, and parsing the corresponding tier to each facility electricity demand in the database. The electricity demand for a given health service level was assumed to remain the same across all countries. The total electricity consumption for the healthcare facilities is calculated by:

(Equation 1) $$Cons_{health,n}={\left[C_{i}\text{∗}d_{i}\right]}_{n}\;$$

where:

**Table 2:** Electricity demand of health facility per level of services category

Health services level	Energy demand [kWh/year]	Electricity tier	Description
Tertiary referral hospital	91,250	tier 5 – full access	Coverage for >120 beds. High-energy requirements. May contain sophisticated diagnostic devices requiring additional power and perform surgical procedures.
Secondary hospital	14,600	tier 4– advanced access	Coverage for 60–120 beds. Moderate energy requirements. May accommodate sophisticated diagnostic medical equipment.
First hospital	7,300	tier 3 – intermediate access	Coverage for 30–60 beds. Low/moderate energy requirements.
Primary health post	1,825	tier 2 – basic access	No beds other than for emergencies/ maternity care. Typically located in a remote setting with limited services and a small staff. Typically operates weekdays. Low energy requirements.

Source: Moner-Girona et al.,^57^ Maina et al., ^63^ USAID,^75^ and AFREA^76^

*n*: identified health facility

*i*: health facility category

*c*_*i*_: daily electricity consumption for category *i* [kWh/day]

*d*_*i*_: number of operational days per category *i* [days]

### Assessment of electrification costs

The cost of providing renewable electricity to each health facility by a standalone PV and battery system is analyzed in two ways: (1) the capital expenditures (CAPEX) based on the system specification for a given location and (2) the corresponding LCOE, a measure of the average net present cost of electricity generation for a generating plant over its lifetime.

#### Costs breakdown

Based on the field installation costs of PV systems in SSA,^40^ we grouped the initial investment costs into three main factor groups for the hardware components and an additional one for the engineering and soft costs (see Table 3). The component costs and their shares are extracted using the bottom-up gathering methodology used by Moner-Girona et al.^40^

**Table 3:** Summary of cost factor groups for off-grid PV systems

#	Factor group	Components
1	PV array	PV modules
		PV mounting structure
2	Balance of system (BOS)	PV cabling
		PV earthing
		charge controller
		DC protections board
		inverter
		AC protections
		AC cabling
3	Storage and monitoring	battery bank and rack
		DC battery protections
		DC battery cabling
		control and battery room
		monitoring board and Software
4	Engineering and soft costs	installation, civil works and miscellaneous materials
		system design and project management
		training
		permitting fees, taxes, and financing
		transport
	Other equipment (for operation and maintenance)	spare parts and storage

Source: adapted from Moner-Girona et al.^40^

In this study, the engineering and soft costs were calculated using the cost shares of each factor group as a percentage of the total cost,^40^ as the financial costs are highly dependent on the country and local conditions, in particular, for accessing remote or difficult to reach locations. The share of total capital costs used was 20% on an average for the PV array and mounting structure, 20% for BOS, 27% for storage and monitoring, and 33% for the engineering and soft costs.

#### Levelized cost of electricity

**Figure 14:**
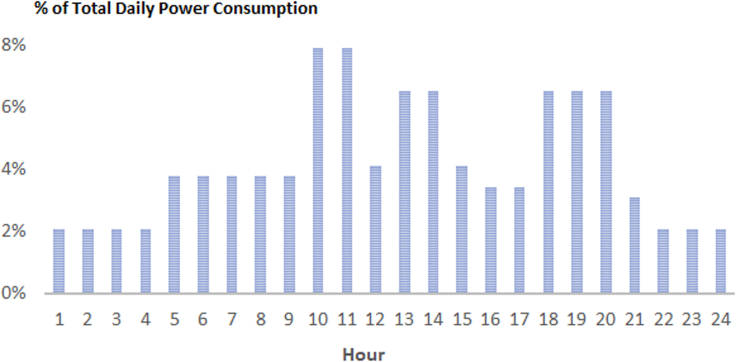
Daily electricity demand profile for healthcare facilities (high daytime consumption) given as hourly % values of the total daily power consumption

The LCOE was estimated for each facility by a location-specific analysis of the energy output and reliability of the PV system, as described by Huld et al.^36^ The PV array and battery storage sizes are optimized for each location to ensure the least-cost solution with a power outage on less than 5% of days in a year. This criterion reflects the fact that the grid reliability in many countries is low, and over one-third of households have a connection that works half or less of the time.^77^ In view of this, the 5% daily outage frequency should provide a quality of service, at least as good as that of the national grid, while keeping the system cost at a competitive level. The PV performance is calculated from location-specific hourly solar radiation values derived from satellite data, supplemented with surface temperature and wind speed data from climate reanalysis.^78^ The daily electricity demand load profile is as defined in the study conducted by Huld et al.,^36^ scaled to match the postulated annual energy demand of a given facility (see Figure 14). The PV module and battery performance algorithms incorporate measured data on PV module and battery performance using Li-ion batteries.^36^

The LCOE was computed for each health facility according to Equation 2, considering solar resource,^78^ the electricity demand for the relevant health facility type (Table 2; Figure 14), together with initial investment costs, CAPEX, replacements, and operation, and maintenance for an operational lifetime of 20 years for the PV array and 10 years for the battery.^57^ The system capital costs are based on field specific data presented in the study conducted by Moner-Girona et al.^40^ The CAPEX_0_ values incorporate the economy of scale effects, with a module price equal to 0.83 EUR/W_p_ and higher prices for systems smaller than 1 kW_p_ (0.99 EUR/W_p_) and lower prices for systems larger than 100 kW_p_ (0.63 EUR/W_p_). The Li-ion battery prices were 350 EUR/kWh nominal when the battery size was smaller than 50 kWh and 280 EUR/kWh when it was larger than 50 kWh. In the case of social energy infrastructure and based on cost-benefit analysis, international institutions^79^^,^^80^ recommend the use of low social discount rates between 3% and 5%, and the latter value is used for all locations.

(Equation 2) LCOEn=CAPEX0+∑t=1T{(Rt+Ot)(1+rn)t}∑t=1T{(ESn)(1+rn)t}

Where:

*n*: identified health facility

$LCOE_{n}$: levelized cost of electricity in facility *n* [EUR/kWh]

$CAPEX_{0}$: initial PV system investment cost at *t* = 0 [EUR]

*t*: time in years *t* = 0 is the installation year

*T*: economic lifetime of the PV system (years)

*O*_*t*_: operation and maintenance cost (2%) in year t (EUR)

*Rt*: replacement cost in year t (EUR)

E_Sn_: average annual electricity production from the given system depending on solar radiation and electricity demand in health center *n* (kWh)

*r*_*n*_: discount rate

**Figure 15:**
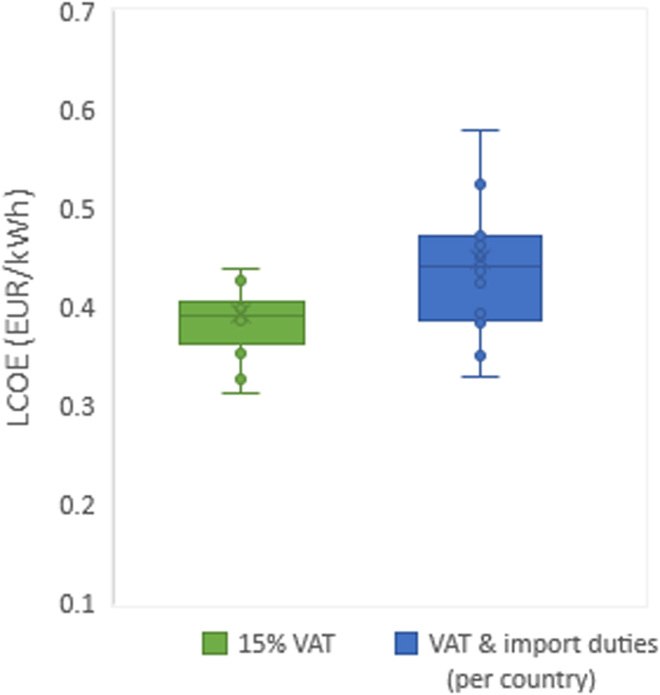
Comparison of levelized cost of electricity computed for health facilities taking into account the particular VAT and import duties per country (in blue) and LCOE calculated without import duties and with a homogeneous 15% VAT (in green). The LCOE values with in-country specific values (blue box) show a higher degree of dispersion (spread box) and higher skewness in the lower values and also higher variability outside the upper and lower quartiles (whiskers).

Import taxes for photovoltaic modules are non-existing or low in most African countries.^81^^,^^82^ This is due to the fact that strategies to support the deployment of renewable energies in Africa are increasing (IRENA, 2020).^1^ Moreover, as the realization of the electrification of health infrastructure is part of a national strategy in collaboration with international organizations, the analysis expects measures to reduce investment risk, in the form of grants (not debt), exemption from VAT (as many countries already have), and exemption from import duties. This is due to the fact that policies tend to favor projects with positive externalities for their overall societal benefits. Figure 15 shows the effect of the variation in VAT and import duties on the LCOE for 16 countries. The LCOE computed for all health facilities, taking into account the particular VAT and import duties per country, shows a higher degree of dispersion (spread), higher skewness in the lower values (blue box), and also higher variability outside the upper and lower quartiles (whiskers). Burundi (high import duty on batteries) and Mozambique (import duties on PV modules and batteries) show the largest differences, with an increase in the LCOE of 0.1 EUR/kWh. 70% of the studied countries show an increase in the LCOE of less than 0.07 EUR/kWh, and Mali and Zambia show no significant difference (due to exemption of VAT and import duties).

#### Adoption of diesel/petrol generators

**Figure 16:**
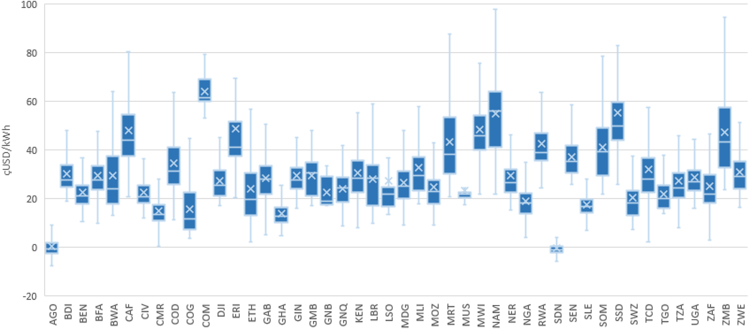
Location-specific estimates of the relative electricity production costs (¢USD/kWh) of solar photovoltaic versus diesel per country Minimum, first quartile, median, third quartile, and maximum of all NEA health centers per country.

The current methodology considers 100% renewable energy systems (due to the mentioned sustainability focus, specifically to reach the SDG7, affordable, reliable, sustainable, and modern energy for all). Despite the fact that the model focuses on sustainable rural electrification, we used location-specific estimates of the relative electricity costs (USD/kWh) of solar photovoltaic versus diesel. The methodology used for this comparison is explained step by step in previous publications,^23^^,^^24^^,^^83^ where the generation cost of electricity that relies on solar photovoltaics is compared with that of diesel. Figure 16 shows the results of the analysis when comparing the differences between the diesel and PV production costs: the minimum, first quartile, median, third quartile, and maximum of all NEA health centers per country. It is observed that for almost all countries, the long-term costs (accounted for 20 years) of diesel are higher than the PV costs, with an average range between 5 and 50 USD cents.

#### Estimating avoided emissions

The estimation of the carbon mitigation potential of using fully renewable mini-grids is based on the avoided GHG emissions in CO2_eq_.^40^ The avoided GHG emissions were calculated by computing the emissions of a standalone diesel generator supplying the same electricity demand over the lifetime of the PV plus battery storage systems. The annual GHG emissions were calculated for 20 years lifetime multiplying the computed emission factor of 1.7 tCO2/MWh for the electricity demand per each health center.

## Data Availability

The electricity grid and the electricity access health facility database (EHFDB) generated during this study are available at: https://data.jrc.ec.europa.eu/collection/id-0076 Visualization (temporal server with beta version) http://d6-dev-africap.jrc.it/energy_tool The methodology subsections were developed to identify the NEA facilities, the population served by each of these facilities (defined as population clusters or catchment areas), and the associated electricity demand. Figure 12 presents a workflow chart summarizing the various stages and data inputs and outputs in the generation and validation of the data.
